# Structural Basis for the Binding of Allosteric Activators Leucine and ADP to Mammalian Glutamate Dehydrogenase

**DOI:** 10.3390/ijms231911306

**Published:** 2022-09-25

**Authors:** Vasily A. Aleshin, Victoria I. Bunik, Eduardo M. Bruch, Marco Bellinzoni

**Affiliations:** 1 Department of Biokinetics, A. N. Belozersky Institute of Physicochemical Biology, Lomonosov Moscow State University, 119234 Moscow, Russia; 2 Department of Biochemistry, Sechenov University, 119048 Moscow, Russia; 3 Faculty of Bioengineering and Bioinformatics, Lomonosov Moscow State University, 119234 Moscow, Russia; 4 Institut Pasteur, Université Paris Cité, CNRS UMR3528, Unité de Microbiologie Structurale, F-75724 Paris, France

**Keywords:** glutamate dehydrogenase, allosteric regulation, leucine, ADP, potassium, acetylation, thiamine triphosphate

## Abstract

Glutamate dehydrogenase (GDH) plays a key role in the metabolism of glutamate, an important compound at a cross-road of carbon and nitrogen metabolism and a relevant neurotransmitter. Despite being one of the first discovered allosteric enzymes, GDH still poses challenges for structural characterization of its allosteric sites. Only the structures with ADP, and at low (3.5 Å) resolution, are available for mammalian GDH complexes with allosteric activators. Here, we aim at deciphering a structural basis for the GDH allosteric activation using bovine GDH as a model. For the first time, we report a mammalian GDH structure in a ternary complex with the activators leucine and ADP, co-crystallized with potassium ion, resolved to 2.45 Å. An improved 2.4-angstrom resolution of the GDH complex with ADP is also presented. The ternary complex with leucine and ADP differs from the binary complex with ADP by the conformation of GDH C-terminus, involved in the leucine binding and subunit interactions. The potassium site, identified in this work, may mediate interactions between the leucine and ADP binding sites. Our data provide novel insights into the mechanisms of GDH activation by leucine and ADP, linked to the enzyme regulation by (de)acetylation.

## 1. Introduction

**Figure 1:**
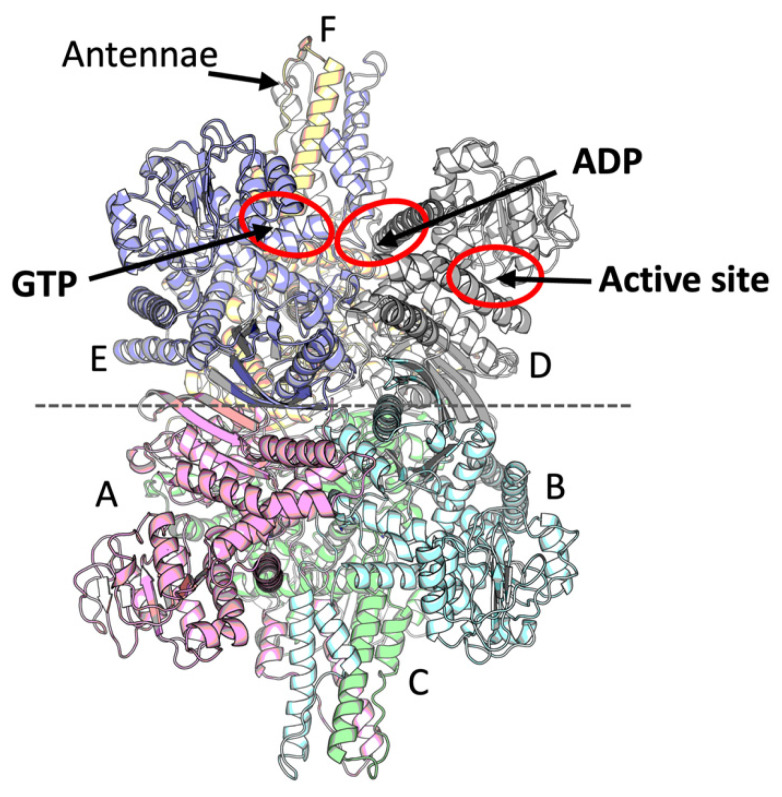
A mammalian GDH model and its ligand binding sites. The six subunits are shown in different colors, while the hexamer is organized as two trimers related by a two-fold axis indicated by a dashed horizontal line. Chains corresponding to 8AR7 and 8AR8 structures are indicated on the graph, showing A, B, C or D, E, F chains to form the two opposite trimers of the GDH hexamer in these models. Two antennae regions are located at the top and bottom of the hexamer, built by three subunits each. Red ovals indicate the active site and allosteric sites for the activator ADP and inhibitor GTP.

Mammalian glutamate dehydrogenase (GDH) is an indispensable metabolic enzyme, particularly relevant for the metabolism of the neurotransmitter glutamate in the brain. Mammalian NAD(P)-dependent GDH of 55 kDa (EC: 1.4.1.3) is significantly different from the pathway-specific NAD- (EC: 1.4.1.2) or NADP-dependent (EC: 1.4.1.4) GDH of non-mammalian species. For example, in *Neurospora crassa*, an NADP-dependent GDH is involved in anabolic processes, while an NAD-dependent GDH functions in glutamate catabolism [1,2]. Nevertheless, with an exception of the family of large 180 kDa GDH [3], most of the NAD- or NADP-specific GDH are homologous to mammalian GDH. The latter enzymes are hexameric, organized as a dimer of trimers (Figure 1), possibly forming complexes/aggregates of higher molecular weight [4,5].

Mammalian GDH is one of the first allosteric enzymes discovered [6]; its allosteric inhibition by GTP and activation by ADP have been known for more than half-a-century, but the structures of respective complexes have been characterized only recently [7,8]. Both ligands possess separate allosteric binding sites (Figure 1). The activation of mammalian GDH by L-leucine and some other hydrophobic amino acids has also been long known [4], yet no structure of the mammalian GDH with leucine has been obtained to date.

Despite more than 20 mammalian GDH structures available in PDB, most of the ligand binding complexes beyond those with the inhibitor GTP are of low resolution. For example, the only two available structures with the GDH activator ADP (1NQT and 6DHK) are at 3.5-angstrom resolution and, most likely, arise from the same diffraction data, which limits the mechanistic understanding of the GDH regulation. The different resolution levels appear to correspond to the two major conformational states of the enzyme—the so-called “open” and “closed” states. These conformations of GDH are well-known from previous kinetic and structural studies [9], extended recently by data from single particle cryo-electron microscopy (cryo-EM) [10]. That is, an open form (in particular, inherent in the apoenzyme or its complex with ADP) has so far been crystallized at medium to low resolution, whereas higher resolution data had been obtained for the closed form (exemplified by complexes with GTP and some apoenzyme structures or complexes with other ligands). The aim of this work is to improve structural characterization of GDH in its open form. We hypothesize that this may be achieved through synergistic action of the GDH allosteric activators, such as ADP [11], leucine [12] and thiamine triphosphate (ThTP) [13], upon their simultaneous presence in a crystallization buffer. Indeed, under such conditions, novel structures of GDH with its activators have been resolved, using GDH from bovine liver as a model of the mammalian GDH family.

We report here the identification of the leucine binding site in a novel ternary complex of mammalian GDH with leucine and ADP at 2.45-angstrom resolution, and the improved characterization of the GDH complex with ADP at the resolution of 2.4 Å. The structures are compared to the known complexes of GDH from different species. In addition, a potassium binding center is localized, which interacts with both the leucine and ADP sites. The results strongly improve understanding of mammalian GDH regulation by allosteric activators and acetylation of the corresponding allosteric sites.

## 2. Results

To enhance the resolution of crystallized GDH complexes with its allosteric activators ADP, ThTP and leucine, we assumed that the enzyme active state may be stabilized by the simultaneous presence of the activators, as each of the activators may contribute to shifting the enzyme conformational ensemble to the open conformation. Hence, we undertook crystallization trials using the activator combinations, in addition to the conditions comprising single activators. As a result, novel binary and ternary complexes of the enzyme with ADP and leucine are crystallized in the media with ThTP, even though ThTP is not identified as a bound ligand in any of the collected datasets.

### 2.1. Identification of the Leucine Binding Site of Mammalian GDH

**Figure 2:**
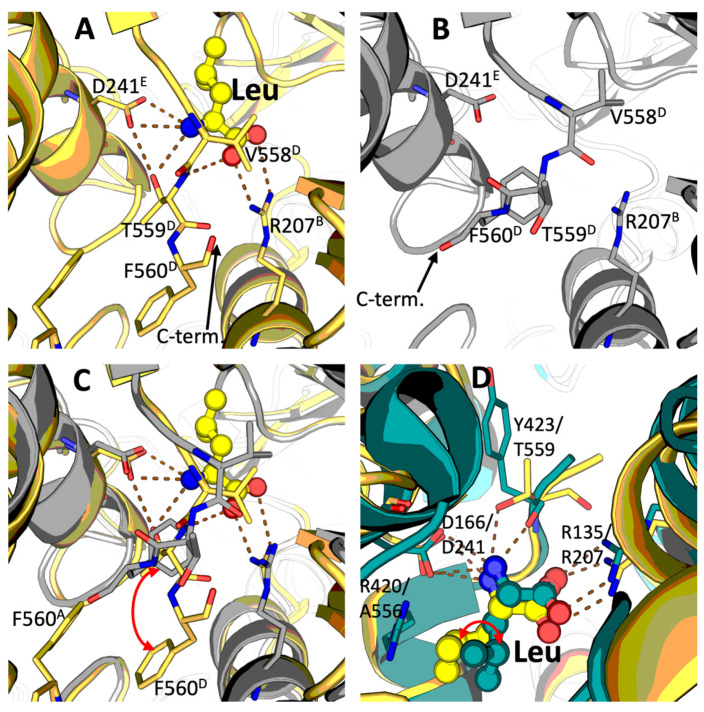
Binding of leucine by bovine GDH. The superscripts of the indicated residues denote belonging of the residues to one of the six monomers within the lower (A, B, C) and upper (D, E, F) trimers, as shown in Figure 1. (**A**)—Structure of leucine (Leu) binding site in the ternary GDH complex with leucine and ADP (PDB 8AR7; the cartoon and carbon atoms are in yellow, while dashed lines indicate the H-bonds). (**B**)—Structure of leucine binding site in the binary complex of GDH with ADP (PDB 8AR8; the cartoon and carbon atoms are in gray). The leucine binding site is shown at the interface of chains B, D and E of the GDH hexamer, as defined in Figure 1. The chain D is traced until residue F560 in both complexes; in the binary GDH∙ADP complex, the GDH C-terminal end assumes an equivalent conformation to the one observed in other GDH structures in the open form (e.g., PDB 3JCZ or 7VDA [10,14]). (**C**)—Conformational changes in the leucine binding site upon binding of the activator (images (**A**) and (**B**) are superposed with the main shift of F560 indicated by the red arrow). (**D**)—Comparison of the leucine binding sites in the bovine (yellow) and *T. thermophilus* (teal) GDH structures. The different conformation of the leucine side chain in the complexes of bacterial and mammalian GDH is indicated by the red arrow. Chain A of PDB 3AOE (GdhB) is used for the bacterial GDH model. The conformation of bound leucine in other subunits, including those encoded by *gdhA*, is similar [15].

For the first time, the structure of a mammalian GDH in a ternary complex with ADP and leucine is solved (Supplementary Figure S1), showing the structural basis of leucine regulation (Figure 2A). Full information on the structural parameters of the complex is provided in Section 4.3 of Materials and Methods. Crystals of bovine GDH in a complex with leucine and ADP (PDB ID: 8AR7) and with ADP alone (PDB ID: 8AR8) are obtained from the protein solution supplemented with the activators, ThTP included. 

A bound leucine molecule is observed in all the six equivalent sites within the mammalian GDH hexamer. Throughout the text, belonging of the considered residues to the monomers A, B, C, D, E, F, shown in Figure 1, is defined by the corresponding superscripts. Leucine binds at the contact area between the three different subunits, involving those from the opposite trimers forming the GDH hexamer. That is, the leucine binding site shown in Figure 2 is formed by the subunits B, E and D (Figure 1). While the R207^B^ guanidinium group makes a salt bridge with the leucine carboxyl group, the D241^E^ carboxyl group from another subunit binds the leucine amino group, which in turn interacts also with the hydroxyl group of T559^D^ located at the C-terminal end of the third subunit. Simultaneously, the main chain amide group of the same T559^D^ is hydrogen-bonded to one of the leucine carboxyl oxygens (Figure 2A). Additionally, the hydroxyl group of T559^D^ acts as an H-bond donor to D241^E^, contributing to a proper orientation of its carboxyl group towards the α-amino group of the bound leucine. Remarkably, while the side chains of D241^E^ and R207^B^ occupy the same position in the absence and presence of leucine (Figure 2B), the conformational change induced by leucine binding essentially involves the C-terminal segment of GDH (the residues 558–560). In the absence of leucine, this C-terminal part is mostly disordered and could be traced for chain D only. In the complex with leucine, where T559^D^ interacts with the amine nitrogen of the bound ligand and D241^E^ (Figure 2A–C), T559^D^ flipping causes its side chain to replace the phenyl group of F560^D^. The ensuing conformation of the aromatic side chain of F560^D^, which moves ~ 4 Å down upon the leucine binding, involves F560^D^ in stacking interactions with the equivalent F560^A^ of the subunit A of the opposite GDH trimer (Figure 2C). The side chain of V558^D^ is partially displaced by leucine upon their hydrophobic interaction (Figure 2 A–C). As a result, there are significant movements and conformational stabilization of the C-terminal residues V558^D^, T559^D^ and F560^D^ upon the leucine binding (Figure 1, Figure 2C and Figure S2). Stabilizing subunit interactions forming a trimer and between the two trimers (Figure 1 and Figure 2), leucine may thus transduce signals not only within, but also between the trimeric protomers of GDH.

Noteworthy, the leucine binding pocket identified by us in the homohexameric bovine GDH is similar to the one observed in heterohexameric GDH from *Thermus thermophilus* (PDB 3AOE [15,16]) (Figure 2D). The main residues responsible for binding the leucine carboxyl and amino groups are conserved, i.e., R135 and D166 from *T. thermophilus* GDH are structurally equivalent to R207 and D241 in bovine GDH. However, the conformation of the side chain of the bound leucine differs between the bacterial and bovine GDH, most likely as a result of the presence of a bulkier side chain in the C-terminal segment in both subunits of bacterial heterohexamer (R415 in GdhA, R420 in GdhB), absent in the bovine enzyme where the equivalent residue is A556 (Figure 2D). Interestingly, although bovine T559 is not conserved, replaced in *T. thermophilus* by a proline (C-terminal residue both in GdhA and GdhB), the H-bond to the leucine amino group is provided in *T. thermophilus* by the carbonyl oxygen of the preceding tyrosine (either Y418 or Y423 in GdhA or GdhB, respectively), which occupies the equivalent place (Figure 2D).

Thus, our GDH structure, crystallized with leucine and ADP, enables novel understanding of the mechanisms of leucine binding to GDH of mammals.

### 2.2. Novel Conformation of GDH·ADP Binary Complex at 2.40-angstrom Resolution

Resolution of both the ternary GDH·ADP·Leu (2.45 Å) and binary GDH·ADP (2.40 Å) complexes strongly exceeds that of the GDH·ADP structures previously reported (3.5 Å). Our data reveal no significant difference in the bound ADP conformation between the GDH·ADP·Leu and GDH·ADP complexes.

**Figure 3:**
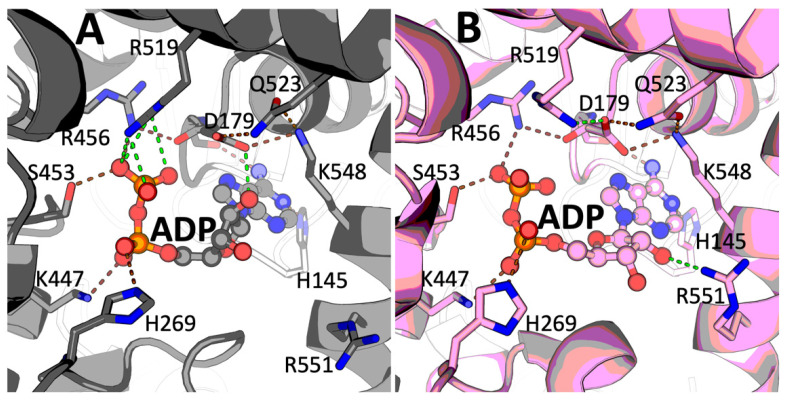
Comparison of ADP binding in the new GDH·ADP complex of 2.4-angstrom resolution (**A**) and previously reported complex 6DHK (**B**). (**A**)—The C2′-endo conformation of the ADP in the 8AR8 structure, shown in gray. (**B**)—The C3′-endo conformation of the ADP in the 6DHK (3.5-angstrom resolution) structure, shown in pink. Similar ADP bonds in both structures are shown by brown dashed lines, and the green lines indicate the differences between the 8AR8 and 6DHK entries.

Of the two GDH structures with ADP available in PDB, namely 1NQT and 6DHK, the 6DHK model (deposited in 2018) is linked to the original paper from 2003 [8], describing the older entry 1NQT by the same authors. We, therefore, use the coordinates from the more recent 6DHK entry as a reference for the comparison to the higher resolution (2.4 Å) GDH·ADP binary complex described here (Section 4.3 of Materials and Methods). Electron density maps in our resolved binary complex unambiguously reveal the bound ADP and the residues involved in ADP binding, such as R519 interacting with the ADP β-phosphate (Figure 3A vs. Figure 3B). The overall conformation of the 8AR8 GDH·ADP complex is very close to 6DHK (the two complexes superimpose with an RMSD of ~ 0.6 Å). However, the conformation of the ADP ribose ring differs. Electron density maps clearly indicate a C2′-endo pucker conformation in our structure for all the ADP molecules bound (Figure 3A, Supplementary Figure S3), in contrast to the previous complex (6DHK) in which either the C2′-endo or the C3′-endo conformation was modeled (Figure 3B). With the higher resolution achieved in our case, the H-bonding between the carboxyl group of D179 and the 2′-hydroxyl of ADP becomes obvious (Figure 3A). In addition, as a consequence of the ribose C2′-endo pucker conformation, we could not observe any interaction between the ADP 2′-hydroxyl and R551, in contrast to the previous models (Figure 3B). 

Thus, our data resolve significant details of ADP conformation bound in the allosteric site of a mammalian GDH.

### 2.3. Identification of the GDH Binding Site for Potassium Ion

**Figure 4:**
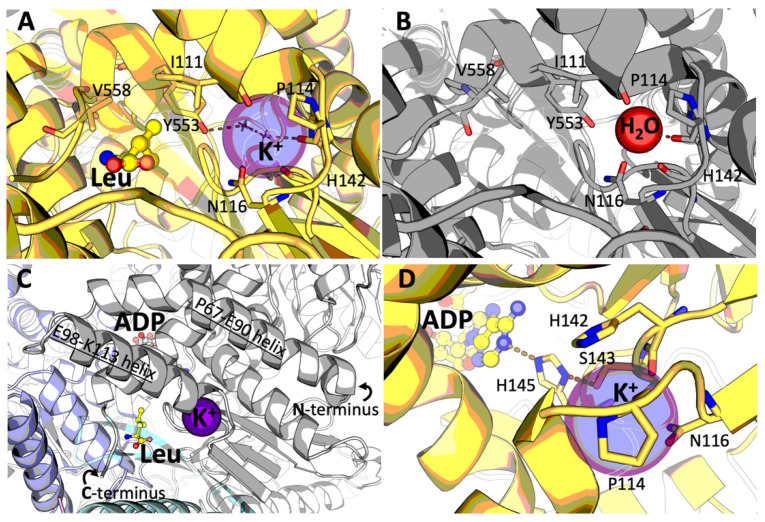
K^+^ binding site in mammalian GDH. (**A**)—The K^+^ binding in the GDH·ADP·Leu complex, colored as in Figure 2A. The interaction of K^+^ and leucine through Y553 and a water molecule from the coordination sphere of K^+^ is shown. (**B**)—The same site in the GDH·ADP complex (colored as in Figure 2B), where the potassium ion is modeled to be substituted by water. (**C**)—Relative positions of the leucine, ADP and potassium binding sites at the interface of GDH trimers, colored as in Figure 1. (**D**)—Interaction between the K^+^ and ADP binding sites. The ADP binding modes are equivalent in both the GDH·ADP·Leu and the GDH·ADP complexes described here. H-bonds are shown as dashed lines. All indicated residues belong to one chain.

The ternary complex of mammalian GDH with ADP and leucine (8AR7) also shows a potassium ion (K^+^) bound in each GDH subunit (Figure 4A). K^+^ is identified as the most likely bound ion from those added to the crystallization mixture (2 mM potassium ADP and 500 mM NaCl) by careful analysis of the electron density, nature and interatomic distances to protein atoms (Supplementary Figure S4A). The presence of orders of magnitude higher concentration of Na^+^ (500 mM) vs. K^+^ (2 mM) in the crystallization medium suggests a specificity for K^+^ in this site, which has not been reported so far for a mammalian GDH. Noteworthy, no ion is unambiguously identified in this site in the binary GDH·ADP complex, crystallized in the presence of Ca^2+^ (20 mM), in addition to K^+^ (2 mM) and Na^+^ (500 mM) (see chapters 4.1 and 4.2 of “Materials and Methods”). The presence of a less significant peak in difference density maps (Supplementary Figure S4B) argued against the presence of either K^+^ or Ca^2+^ at significant occupancy. Considering the shorter interatomic distances to protein atoms and the incomplete coordination spheres in most chains, we eventually modeled the species bound to the same site as water (Supplementary Figure S4B), although the presence of a lighter cation such as Na^+^, or a mixed situation including the alternate presence of Na^+^ and/or K^+^ at lower occupancies, cannot be ruled out.

The K^+^ binding site is located at the end of the α-helix which ends with P114 and is formed by the main chain carbonyl atoms of I111, I112, P114 and H142 and the carboxamide oxygen of N116 (Figure 4). The α-helix near the cation binding site interacts with the C-terminal GDH helix (Figure 4C). Taking into account the significant involvement of the C-terminus of the enzyme in leucine binding, the substitution of the water molecule in the GDH·ADP complex by a potassium ion in the GDH·ADP·Leu complex may be due to the leucine-induced rearrangement of the C-terminal GDH part. Remarkably, H142 participates directly in the K^+^ site formation through its main chain oxygen, while S143 links the cation to the ADP site through H145, whose imidazole side chain in turn H-bonds both to S143 and to the ADP adenine ring (Figure 4D). In addition, a water molecule from the K^+^ coordination sphere is H-bonded to the hydroxyl group of Y553, whose side chain makes Van der Waals interactions with the bound leucine (Figure 4A). As a result, the newly identified binding of K^+^ may stabilize the open conformation of the enzyme in the ternary GDH·ADP·Leu complex, transducing signals between the ADP and leucine sites. Our structural data suggest that the affinity to K^+^ may be significantly decreased in the binary GDH·ADP complex.

## 3. Discussion

We report here new structures of mammalian GDH complexes with the allosteric activators ADP and leucine. The structures show the open form of the enzyme, where the GDH active sites are easily accessible to the substrates, under significantly increased (down to 2.4 Å) resolution, compared to the previously known structures of the GDH open form with ADP (3.5 Å). While the structures of bacterial or fungal GDHs have been deposited at resolutions down to 1.7 Å (PDB 5GUD and 7ECR) [17,18], the lower resolution of mammalian GDH in X-ray crystallography correlates with the presence of a 50-residue “antenna” domain, proposed to be an evolutionary acquisition for interactive regulation of the GDH activity by its multiple allosteric ligands [9]. Although single particle cryo-EM has been used to overcome the limitation, cryo-EM complexes of mammalian GDH with either GTP or NADH have only been reported at a medium resolution (3.3 Å) [10]. On the other hand, the highest resolution cryo-EM model (1.8 Å) available so far for bovine GDH (PDB 5K12) is of limited use, as it does not show most of the NAD(P)(H) binding domains nor significant parts of the N- and C-terminal ends [19].

### 3.1. Leucine Binding Site

The GDH·ADP·Leu ternary complex resolved in this work represents the first structure of the leucine allosteric site in mammalian GDH. The only previously reported structure of this allosteric site used a GDH with 50 kDa from *T. thermophilus*, a Gram-negative thermophile bacterium possessing a GDH heterohexamer formed by four GdhA and two GdhB subunits [15,16]. Such heteromeric organization of GDH, where one type of the subunits is catalytically inactive and acts as the regulatory subunits for activation by leucine (GdhA) [20], is not known in mammals. Additionally, the GDH from *T. thermophilus* does not bind ADP, but instead may be activated by AMP within its heterocomplex with the product of the gene *TTC1249*, proposed to act as an AMP-sensory subunit [20]. Despite these significant differences in the genetic basis of the GDH regulation, the leucine binding sites of bovine and *T. thermophilus* GDH are very similar, suggesting a significant evolutionary conservation of the protein structural elements providing for the allosteric activation of GDH.

Remarkably, the site-directed mutagenesis of human GDH2 has revealed that R151M and D185A mutants (the numbering does not include the N-terminal transit peptide 1–53), substituted in positions equivalent to the bovine GDH R207 and D241 that are essential for leucine binding (Figure 2), are not activated by leucine [15]. These findings are consistent with our structural data, confirming functional significance of the mammalian GDH residues interacting with leucine at the allosteric site.

### 3.2. Potassium Ion Binding Site

The K^+^ binding site of mammalian GDH, that is described here, is structurally equivalent to the K^+^ site identified in GDH1 from *Arabidopsis thaliana* (6YEH [21]) and to one of the multiple Na^+^ binding sites in the structure of human GDH2 (6G2U [22]) (Figure 4). Among bacterial GDH with the resolved structures, only GDH from *Corynebacterium glutamicum* contains multiple sites for potassium ion binding (5GUD [17]), one of them coinciding with the K^+^ site of mammalian GDH identified by us. Such conservation of this cation binding site across different species implies its biological significance, which is to be established in further studies. Our structural data provide hints about a high specificity of this site to potassium ion in the ternary GDH·ADP·Leu complex, where K^+^ is observed to bind in the presence of a 250-fold molar excess of Na^+^. However, in the binary GDH·ADP complex, crystallized in the presence of the same concentrations of potassium and sodium ions, no potassium ion is bound to the site. The data suggest a role of the bound K^+^ in stabilizing the open conformation of GDH once activated by leucine. In view of the known complexity and potential synergism of the regulatory action of monovalent and divalent cations [23], identification of a specific K^+^ site may add new mechanistic insights to the data on GDH regulation by Zn^2+^ in mammals [24,25,26] or by Ca^2+^ in plants [21].

### 3.3. Implications of the ADP and Leucine Binding for the GDH Regulation by Acetylation

Despite the available structural data on mammalian GDH, our understanding of the functional significance of the reported multiple post-translational acylations of GDH lysines remains limited. The role of acetylation of K503 in the allosteric GTP site of the rat brain GDH for the GTP inhibition has been predicted from the structure of the inhibitory complex [27]. New structural insights in the binding of the GDH activators ADP and leucine provide for a better understanding of the acylation-imposed regulation of the allosteric effects. Of the bovine GDH residues involved in ADP binding, K548 (Figure 3) corresponds to human/mouse K545 (Supplementary Figure S5), known to be acetylated [28,29]. As Figure 3A shows, this residue helps orienting D179 for its interaction with the 2′-hydroxyl of ADP. Changing the charge of the ε-amino group and introducing steric constraints, acetylation of K545/548 is thus expected to decrease the ADP binding affinity.

Of the bovine GDH lysine residues proximal to the newly identified leucine binding site, K203 corresponds to the acetylatable human/mouse K200 [28,29]. As K203 is located close to the C-terminal end of GDH, the K203 acetylation may change conformational mobility of the GDH C-terminus, affecting allosteric action of leucine (Figure 2). 

Thus, localization of the leucine site and improved understanding of the ADP binding provide new information for unraveling functional consequences of acylations of the GDH lysine residues.

### 3.4. Binding of Thiamine Derivatives

Although both GDH complexes described here have been obtained in the presence of ThTP, which is a known activator of GDH [13], no bound ThTP could be detected in our crystals. The failure to see ThTP bound to GDH may be ascribed to several factors, not last the long time required to grow suitable bovine GDH crystals, causing ThTP hydrolysis and/or degradation due to the thiazolium ring opening at pH > 7. Nevertheless, it is worth noting that both crystal forms of GDH in its active open form, obtained here in the presence of ThTP, have not been reported before, neither could the properly diffracting samples be obtained in our crystallization mixtures in the absence of ThTP or with ThTP replaced by thiamine diphosphate. Our study, therefore, favors a contribution of ThTP to the conformational stabilization of the activated form of GDH during crystallization. Along with a tight relationship between the GDH reaction and the thiamine-dependent mitochondrial dehydrogenases of 2-oxoacids [30], the observations warrant further studies on the ThTP-dependent regulation of GDH.

## 4. Materials and Methods

### 4.1. Reagents

Bovine GDH (Merck, G7882) was purchased as lyophilized powder, dissolved in 50 mM HEPES with 500 mM NaCl solution, pH 7 and used for crystallization as it is at 30 mg/mL concentration. ADP (Merck, A5285) was used in the form of monopotassium salt (K·ADP). L-leucine (Merck, L8000) was used. ThTP was synthesized according to the previously published procedure [31] and its 95% purity was checked by NMR. The buffers, salts and other reagents were from Merck.

### 4.2. Crystallization and Data Collection

Crystallization was carried out as screenings at 4 °C temperature using the sitting-drop vapor diffusion method and a Mosquito nanoliter-dispensing crystallization robot (TTP Labtech), according to established protocols at the Institut Pasteur Crystallography Facility [32]. Crystals appeared after variable times (1 to 2 months). Optimized conditions for crystal growth of the different protein complexes were as follows: (GDH·ADP·Leu): 20% EtOH, 30% 2-methyl2,4-pentanediol (MPD), with a GDH solution (30 mg/mL) supplemented with 2 mM monopotassium ADP, 2 mM trisodium ThTP and 10 mM L- leucine (pH 7); (GDH·ADP): 20 mM CaCl_2_, 100 mM Na-acetate buffer (pH 4.6), 30% MPD, with a GDH solution (30 mg/mL) supplemented with 2 mM trisodium ThTP and 2 mM monopotassium ADP. Since both crystallization conditions contained 30% MPD, crystals were frozen in liquid nitrogen without addition of cryo-protectants. X-ray diffraction data were collected from single crystals at 100 K using synchrotron radiation at the beamline ID30B (ESRF, Grenoble, France).

### 4.3. Structure Determination and Refinement

The data were processed with XDS (version Jan 2022) [33] run through autoPROC 1.0.5 [34] and scaled with STARANISO 2.3.87, provided within the same software, to properly account for diffraction anisotropy. The structures were solved by molecular replacement through the program PHASER [35]. Coordinates of bovine GDH [PDB: 3JCZ] in unliganded open form (from single particle cryo-EM [10]) were used as the search model to first solve the structure of GDH in apo form, which, in turn, served as the molecular replacement search model for the following datasets. Manual rebuilding, ligand pose and adjustments of the models were performed with COOT 0.9.6 [36]. Refinement was carried out with BUSTER 2.10.4 (Global Phasing Ltd.), applying local structure similarity restraints for non-crystallography symmetry [37] and a Translation–Libration–Screw (TLS) model. Validation of models was performed with MolProbity [38] and the validation tools in PHENIX 1.20.1 [39]. ‘Polder’ omit maps were computed by the phenix.polder tool [40], and assignments of bound cations were checked through the ‘CheckMyMetal’ server [41]. A summary of data collection and refinement statistics is provided in Table 1. Graphical representations were rendered with PyMOL 2.5.0 (Schrödinger LLC).

Resolution limits were determined by applying an anisotropic cut-off via STARANISO 2.3.87, part of the autoPROC 1.0.5 data processing software [34]; data in parentheses refer to the highest resolution shell.

^a^R_pim_ = Σ_hkl_ [1/(N-1)]^1/2^Σ_i_|I_i_(hkl) − ⟨I⟩(hkl)|/Σ_hkl_ Σ_i_ I_i_(hkl), where N is the multiplicity, I_i_ is the intensity of reflection i and ⟨I⟩(hkl) is the mean intensity of all symmetry-related reflections [42].

^b^R_work_ = Σ||F_o_| − |F_c_||/Σ|F_o_|, where F_o_ and F_c_ are the observed and calculated structure factor amplitudes. Five percent of the reflections were reserved for the calculation of R_free_.

^c^Calculated with MolProbity [38] within the autoBUSTER refinement suite.

### 4.4. Multiple Sequence Alignment

The GDH sequences were aligned with T-Coffee [43], and the resulting alignment was generated with Jalview 2 [44].

## Figures and Tables

**Table 1:** Summary of the X-ray data.

	GDH·ADP·Leu	GDH·ADP
Synchrotron beamline	ESRF ID30B	ESRF ID30B
Space group	P2_1_	P1
Unit-cell parameters		
a, b, c (Å)	90.88, 178.71, 123.88	87.51, 92.03, 119.57
α, β, γ (°)	90, 104.00, 90	99.35, 106.73, 109.73
Resolution range (Å)	120.20–2.45 (2.79–2.45)	47.46–2.40 (2.48–2.40)
Wavelength (Å)	0.9763	0.9763
No. measured reflections	445,692	331,373
No. unique reflections	82,997	96,056
Multiplicity	5.4 (5.7)	3.4 (3.6)
Completeness (%)	92.0 (60.9)	89.9 (86.0)
Average I/σ(I)	6.8 (1.8)	5.0 (2.0)
Rpim^a^	0.094 (0.551)	0.175 (0.398)
CC(1/2)	0.989 (0.407)	0.925 (0.668)
**Refinement statistics**		
R_work_^b^ (%)	19.2	20.4
R_free_^b^ (%)	21.4	23.6
No. of non-H atoms		
Macromolecule	22994	22937
Ligands/ions	222	162
Water molecules	439	1019
Average B-factors	68.9	41.4
Rms deviations^c^		
Bonds (Å)	0.010	0.010
Angles (°)	1.37	1.27
**Molprobity statistics**		
Clashscore	4.26	4.30
Ramachandran outliers (%)	0.07	0.00
Ramachandran favored (%)	97.41	98.6
Rotamer outliers (%)	3.24	3.61
C-beta deviations	0	1
PDB entry code	8AR7	8AR8

## Data Availability

The atomic coordinates and structure factors of the two crystal structures were deposited in the PDB with codes 8AR7 (GDH·ADP·Leu) and 8AR8 (GDH·ADP). All other data needed for evaluation of the conclusions are present in the paper.

## Supplementary Materials

The following supporting information can be downloaded at: https://www.mdpi.com/article/10.3390/ijms231911306/s1, Figure S1. Binding poses of the allosteric regulators ADP and leucine in the ternary GDH·ADP·Leu complex (PDB 8AR7); Figure S2. cartoon view of the GDH hexamer in ternary complex with ADP and Leu (PDB 8AR7); Figure S3. superimposition of ADP bound to bovine GDH in the previously reported complex at 3.5-angstrom resolution (PDB 6DHK, cyan) and in the binary complex here described at 2.4-angstrom resolution (PDB 8AR8, green/yellow); Figure S4. detailed view of the K^+^-binding site; Figure S5. multiple alignment of the five mentioned GDH sequences.

## Abbreviations

GDH	glutamate dehydrogenase
ThTP	thiamine triphosphate
MPD	2-methyl2,4-pentanediol
Cryo-EM	cryo-electron microscopy

## References

[1] Barratt R.W., Strickland W.N. (1963). Purification and characterization of a TPN-specific glutamic acid dehydrogenase from Neurospora crassa. Arch. Biochem. Biophys..

[2] Veronese F.M., Nyc J.F., Degani Y., Brown D.M., Smith E.L. (1974). Nicotinamide Adenine Dinucleotide-specific Glutamate Dehydrogenase of Neurospora. J. Biol. Chem..

[3] Lázaro M., Melero R., Huet C., López-Alonso J.P., Delgado S., Dodu A., Bruch E.M., Abriata L.A., Alzari P.M., Valle M. (2021). 3D architecture and structural flexibility revealed in the subfamily of large glutamate dehydrogenases by a mycobacterial enzyme. Commun. Biol..

[4] Yielding K.L., Tomkins G.M. (1961). An Effect of L-Leucine and Other Essential Amino Acids on the Structure and Activity of Glutamic Dehydrogenase. Proc. Natl. Acad. Sci. USA.

[5] Frieden C. (1962). The Effect of pH and Other Variables on the Dissociation of Beef Liver Glutamic Dehydrogenase. J. Biol. Chem..

[6] Talal N., Tomkins G.M. (1964). Allosteric Properties of Glutamate Dehydrogenases from Different Sources. Science.

[7] Peterson P.E., Smith T.J. (1999). The structure of bovine glutamate dehydrogenase provides insights into the mechanism of allostery. Structure.

[8] Banerjee S., Schmidt T., Fang J., Stanley C.A., Smith T.J. (2003). Structural studies on ADP activation of mammalian glutamate dehydrogenase and the evolution of regulation. Biochemistry.

[9] Smith T.J., Stanley C.A. (2008). Untangling the glutamate dehydrogenase allosteric nightmare. Trends Biochem. Sci..

[10] Borgnia M.J., Banerjee S., Merk A., Matthies D., Bartesaghi A., Rao P., Pierson J., Earl L.A., Falconieri V., Subramaniam S. (2016). Using Cryo-EM to Map Small Ligands on Dynamic Metabolic Enzymes: Studies with Glutamate Dehydrogenase. Mol. Pharmacol..

[11] Bailey J., Bell E.T., Bell J.E. (1982). Regulation of bovine glutamate dehydrogenase. The effects of pH and ADP. J. Biol. Chem..

[12] Couée I., Tipton K.F. (1989). Activation of glutamate dehydrogenase by l-leucine. Biochim. Et Biophys. Acta (BBA)-Protein Struct. Mol. Enzymol..

[13] Mkrtchyan G., Aleshin V., Parkhomenko Y., Kaehne T., Di Salvo M.L., Parroni A., Contestabile R., Vovk A., Bettendorff L., Bunik V. (2015). Molecular mechanisms of the non-coenzyme action of thiamin in brain: Biochemical, structural and pathway analysis. Sci. Rep..

[14] Fan H., Wang B., Zhang Y., Zhu Y., Song B., Xu H., Zhai Y., Qiao M., Sun F. (2021). A cryo-electron microscopy support film formed by 2D crystals of hydrophobin HFBI. Nat. Commun..

[15] Tomita T., Kuzuyama T., Nishiyama M. (2011). Structural basis for leucine-induced allosteric activation of glutamate dehydrogenase. J. Biol. Chem..

[16] Tomita T., Miyazaki T., Miyazaki J., Kuzuyama T., Nishiyama M. (2010). Hetero-oligomeric glutamate dehydrogenase from Thermus thermophilus. Microbiology.

[17] Tomita T., Yin L., Nakamura S., Kosono S., Kuzuyama T., Nishiyama M. (2017). Crystal structure of the 2-iminoglutarate-bound complex of glutamate dehydrogenase from Corynebacterium glutamicum. FEBS Lett..

[18] Godsora B.K.J., Prakash P., Punekar N.S., Bhaumik P. (2021). Molecular insights into the inhibition of glutamate dehydrogenase by the dicarboxylic acid metabolites. Proteins Struct. Funct. Bioinform..

[19] Merk A., Bartesaghi A., Banerjee S., Falconieri V., Rao P., Davis M.I., Pragani R., Boxer M.B., Earl L.A., Milne J.L.S. (2016). Breaking Cryo-EM Resolution Barriers to Facilitate Drug Discovery. Cell.

[20] Tomita T., Matsushita H., Yoshida A., Kosono S., Yoshida M., Kuzuyama T., Nishiyama M., Galperin M.Y. (2019). Glutamate Dehydrogenase from Thermus thermophilus Is Activated by AMP and Leucine as a Complex with Catalytically Inactive Adenine Phosphoribosyltransferase Homolog. J. Bacteriol..

[21] Grzechowiak M., Sliwiak J., Jaskolski M., Ruszkowski M. (2020). Structural Studies of Glutamate Dehydrogenase (Isoform 1) From Arabidopsis thaliana, an Important Enzyme at the Branch-Point Between Carbon and Nitrogen Metabolism. Front. Plant Sci..

[22] Dimovasili C., Fadouloglou V.E., Kefala A., Providaki M., Kotsifaki D., Kanavouras K., Sarrou I., Plaitakis A., Zaganas I., Kokkinidis M. (2021). Crystal structure of glutamate dehydrogenase 2, a positively selected novel human enzyme involved in brain biology and cancer pathophysiology. J. Neurochem..

[23] Gohara D.W., Di Cera E. (2016). Molecular Mechanisms of Enzyme Activation by Monovalent Cations. J. Biol. Chem..

[24] Adelstein S.J., Vallee B.L. (1958). Zinc in Beef Liver Glutamic Dehydrogenase. J. Biol. Chem..

[25] Wolf G., Schmidt W. (1983). Zinc and glutamate dehydrogenase in putative glutamatergic brain structures. Acta Histochem..

[26] Bailey J., Powell L., Sinanan L., Neal J., Li M., Smith T., Bell E. (2011). A novel mechanism of V-type zinc inhibition of glutamate dehydrogenase results from disruption of subunit interactions necessary for efficient catalysis. FEBS J..

[27] Aleshin V.A., Mkrtchyan G.V., Kaehne T., Graf A.V., Maslova M.V., Bunik V.I. (2020). Diurnal regulation of the function of the rat brain glutamate dehydrogenase by acetylation and its dependence on thiamine administration. J. Neurochem..

[28] Lombard D.B., Alt F.W., Cheng H.L., Bunkenborg J., Streeper R.S., Mostoslavsky R., Kim J., Yancopoulos G., Valenzuela D., Murphy A. (2007). Mammalian Sir2 homolog SIRT3 regulates global mitochondrial lysine acetylation. Mol. Cell. Biol..

[29] Lundby A., Lage K., Weinert B.T., Bekker-Jensen D.B., Secher A., Skovgaard T., Kelstrup C.D., Dmytriyev A., Choudhary C., Lundby C. (2012). Proteomic analysis of lysine acetylation sites in rat tissues reveals organ specificity and subcellular patterns. Cell Rep..

[30] Mkrtchyan G.V., Graf A., Trofimova L., Ksenofontov A., Baratova L., Bunik V. (2018). Positive correlation between rat brain glutamate concentrations and mitochondrial 2-oxoglutarate dehydrogenase activity. Anal Biochem..

[31] Bettendorff L., Nghiêm H.-O., Wins P., Lakaye B. (2003). A general method for the chemical synthesis of γ-32P-labeled or unlabeled nucleoside 5′-triphosphates and thiamine triphosphate. Anal. Biochem..

[32] Weber P., Pissis C., Navaza R., Mechaly A.E., Saul F., Alzari P.M., Haouz A. (2019). High-Throughput Crystallization Pipeline at the Crystallography Core Facility of the Institut Pasteur. Molecules.

[33] Kabsch W. (2010). Xds. Acta Crystallogr. Sect. D Biol. Crystallogr..

[34] Vonrhein C., Flensburg C., Keller P., Sharff A., Smart O., Paciorek W., Womack T., Bricogne G. (2011). Data processing and analysis with theautoPROCtoolbox. Acta Crystallogr. Sect. D Biol. Crystallogr..

[35] McCoy A.J., Grosse-Kunstleve R.W., Adams P.D., Winn M.D., Storoni L.C., Read R.J. (2007). Phasercrystallographic software. J. Appl. Crystallogr..

[36] Emsley P., Lohkamp B., Scott W.G., Cowtan K. (2010). Features and development of Coot. Acta Crystallogr. Sect. D Biol. Crystallogr..

[37] Smart O.S., Womack T.O., Flensburg C., Keller P., Paciorek W., Sharff A., Vonrhein C., Bricogne G. (2012). Exploiting structure similarity in refinement: Automated NCS and target-structure restraints inBUSTER. Acta Crystallogr. Sect. D Biol. Crystallogr..

[38] Williams C.J., Headd J.J., Moriarty N.W., Prisant M.G., Videau L.L., Deis L.N., Verma V., Keedy D.A., Hintze B.J., Chen V.B. (2018). MolProbity: More and better reference data for improved all-atom structure validation. Protein Sci..

[39] Liebschner D., Afonine P.V., Baker M.L., Bunkóczi G., Chen V.B., Croll T.I., Hintze B., Hung L.-W., Jain S., McCoy A.J. (2019). Macromolecular structure determination using X-rays, neutrons and electrons: Recent developments in Phenix. Acta Crystallogr. Sect. D Struct. Biol..

[40] Liebschner D., Afonine P.V., Moriarty N.W., Poon B.K., Sobolev O.V., Terwilliger T.C., Adams P.D. (2017). Polder maps: Improving OMIT maps by excluding bulk solvent. Acta Crystallogr. Sect. D Struct. Biol..

[41] Zheng H., Cooper D.R., Porebski P.J., Shabalin I.G., Handing K.B., Minor W. (2017). CheckMyMetal: A macromolecular metal-binding validation tool. Acta Crystallogr. Sect. D Struct. Biol..

[42] Weiss M.S. (2001). Global indicators of X-ray data quality. J. Appl. Crystallogr..

[43] Notredame C., Higgins D.G., Heringa J. (2000). T-coffee: A novel method for fast and accurate multiple sequence alignment 1 1Edited by J. Thornton. J. Mol. Biol..

[44] Waterhouse A.M., Procter J.B., Martin D.M.A., Clamp M., Barton G.J. (2009). Jalview Version 2—A multiple sequence alignment editor and analysis workbench. Bioinformatics.

