# Supplementary material for: Structural Basis for the Binding of Allosteric Activators Leucine and ADP to Mammalian Glutamate Dehydrogenase
Source: Int J Mol Sci. 2022 Sep 25;23(19):11306. doi: 10.3390/ijms231911306 (PMC9570180; doi:10.3390/ijms231911306)
Supplement: Supplementary file 1 [file ijms-23-11306-s001.zip › ijms-1910060-supplementary.pdf]

Structural Basis for the Binding of Allosteric Activators Leucine and ADP to Mammalian Glutamate Dehydrogenase

Vasily A. Aleshin [1,2], Victoria I. Bunik [1,2,3], Eduardo M. Bruch [4], and Marco Bellinzoni [4]

[1] Department of Biokinetics, A. N. Belozersky Institute of Physicochemical Biology, Lomonosov Moscow State University, 119234 Moscow, Russia
[2] Department of Biochemistry, Sechenov University, 119048 Moscow, Russia
[3] Faculty of Bioengineering and Bioinformatics, Lomonosov Moscow State University, 119234 Moscow, Russia
[4] Institut Pasteur, Université Paris Cité, CNRS UMR3528, Unité de Microbiologie Structurale, F-75724 Paris, France.

SUPPLEMENTARY FIGURES

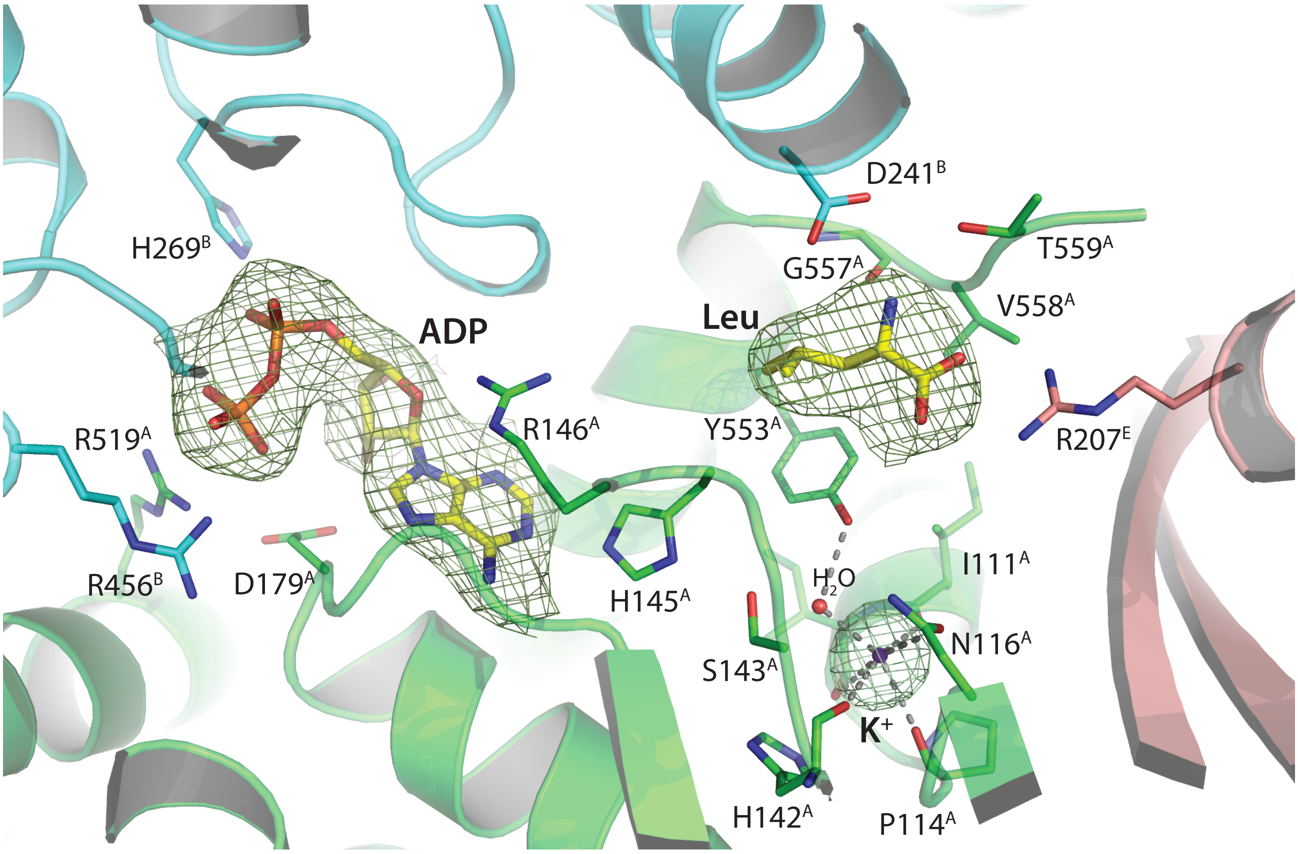

Supplementary Figure S1. Binding pose of the allosteric regulators ADP and leucine in the ternary GDH·ADP·Leu complex (PDB 8AR7). The potassium ion identified nearby the leucine and ADP binding sites is shown as well. The green mesh represents the 'polder' omit map for each ligand (Liebschner *et al.*, Acta Cryst. D, 2017), contoured at the 5σ level. Residues involved in ligand binding are depicted as sticks; colors refer to different protomers.

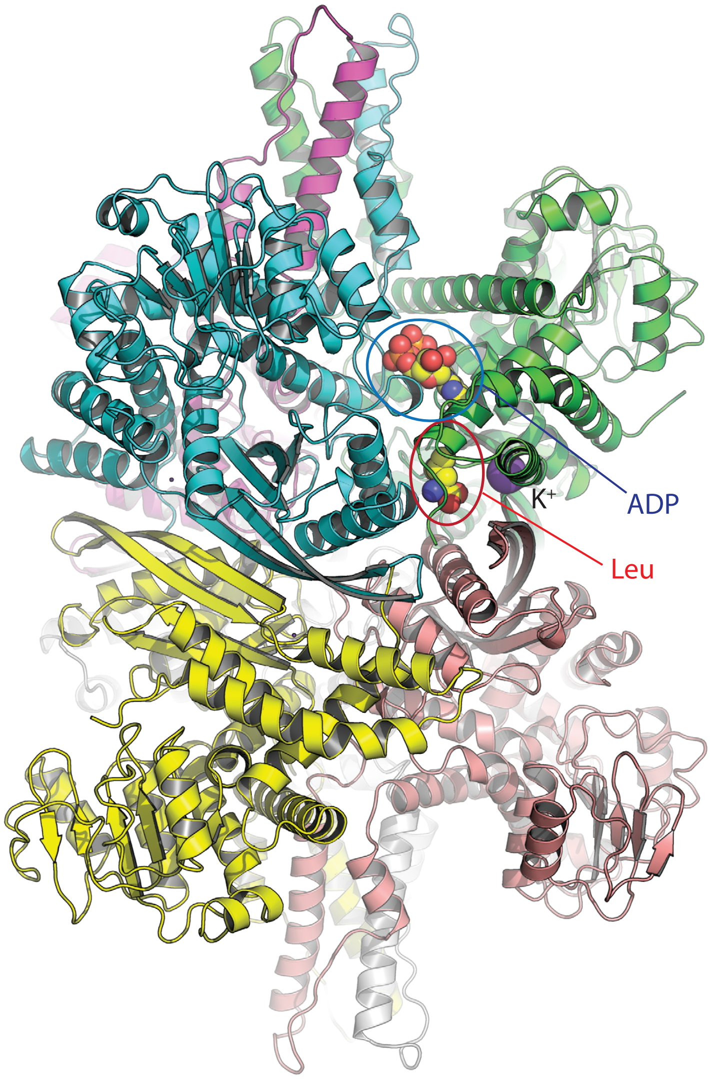

Supplementary Figure S2. Cartoon view of the GDH hexamer in ternary complex with ADP and Leu (PDB 8AR7). ADP, Leu and the bound K^+ are depicted as spheres (for one chain only; indicated). To note the Leu binding site located at the trimer/trimer interface (red ellipse). Colors refer to different monomers.

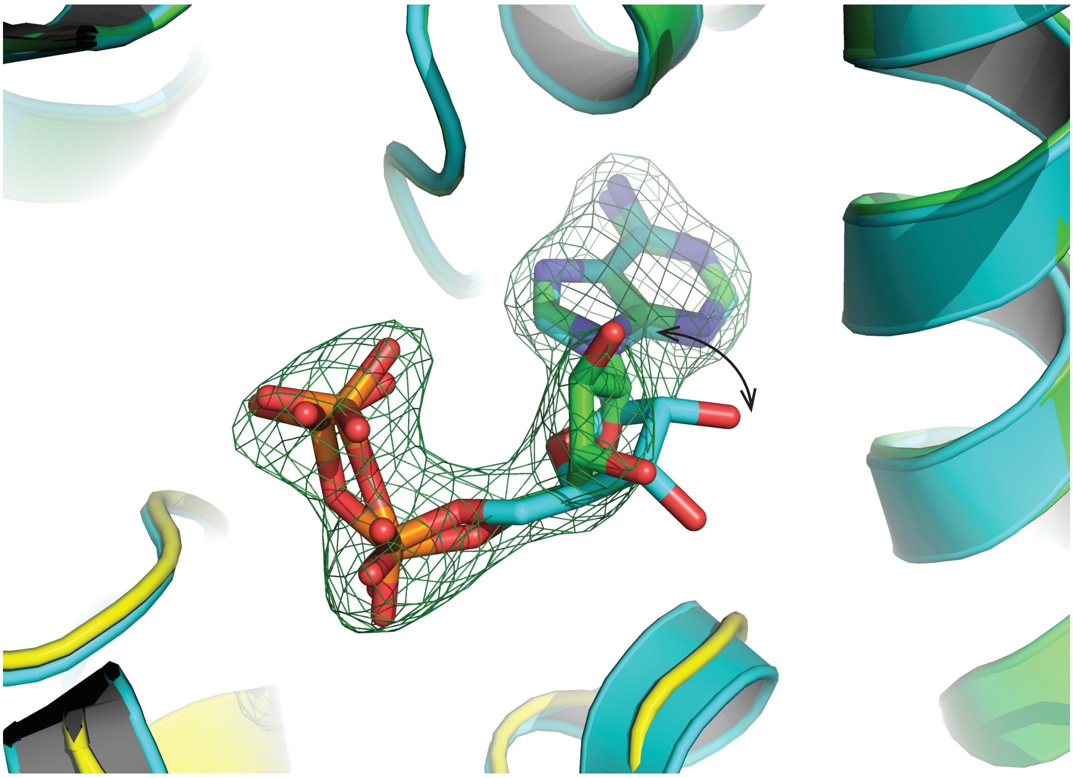

Supplementary Figure S3. Superimposition of ADP bound to bovine GDH in the previously reported complex at 3.5 Å resolution (PDB 6DHK, cyan) and in the binary complex here described at 2.4 Å resolution (PDB 8AR8, green/yellow). The green mesh represents the 'polder' omit map for ADP in 8AR8, contoured at the 5σ level. The arrow highlights the C2'-endo pucker conformation in our structure (green), fully supported by the eletron density, *vs.* the C3'-endo pucker conformation for ADP modelled in 6DHK (cyan). To note, ADP ribose also shows an equivalent C2'-endo pucker conformation in the ternary GDH·ADP·Leu complex described in this work.

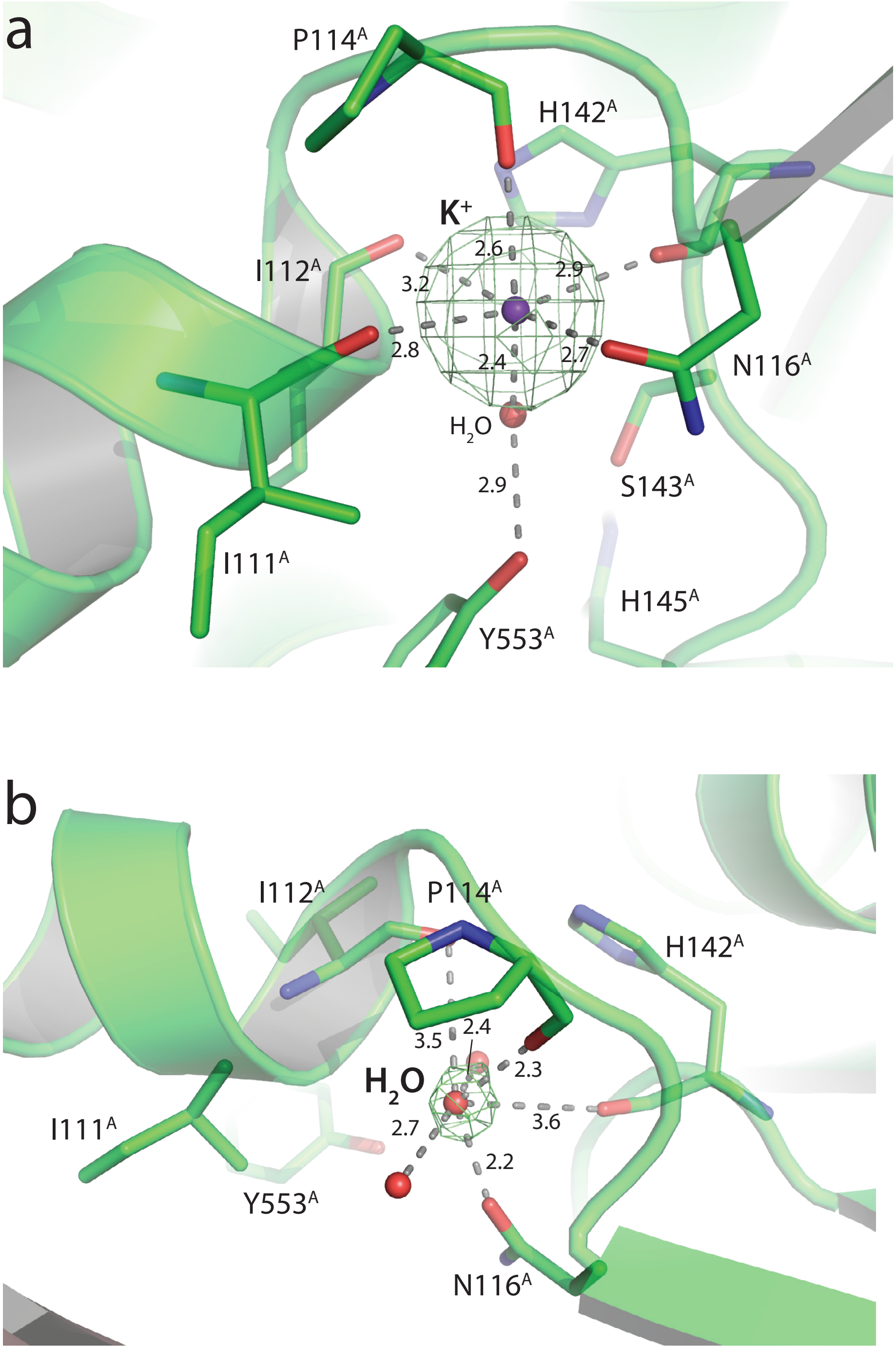

Supplementary Figure S4. (**a**) Detailed view of the K^+-binding site identified in the GDH·ADP·Leu complex (PDB 8AR7, chain A). The green mesh represents the 'polder' omit map for the K^+ cation, contoured at the 5σ level. Grey dashes indicate interatomic distances, with the measured values reported in Å (numerical values aside). (**b**) View of the same site in the GDH·ADP binary complex described in this work (PDB 8AR8, chain A), with a water molecule rather modelled in the same site (green mesh corresponding to the polder omit density at 5σ, as in (a)).

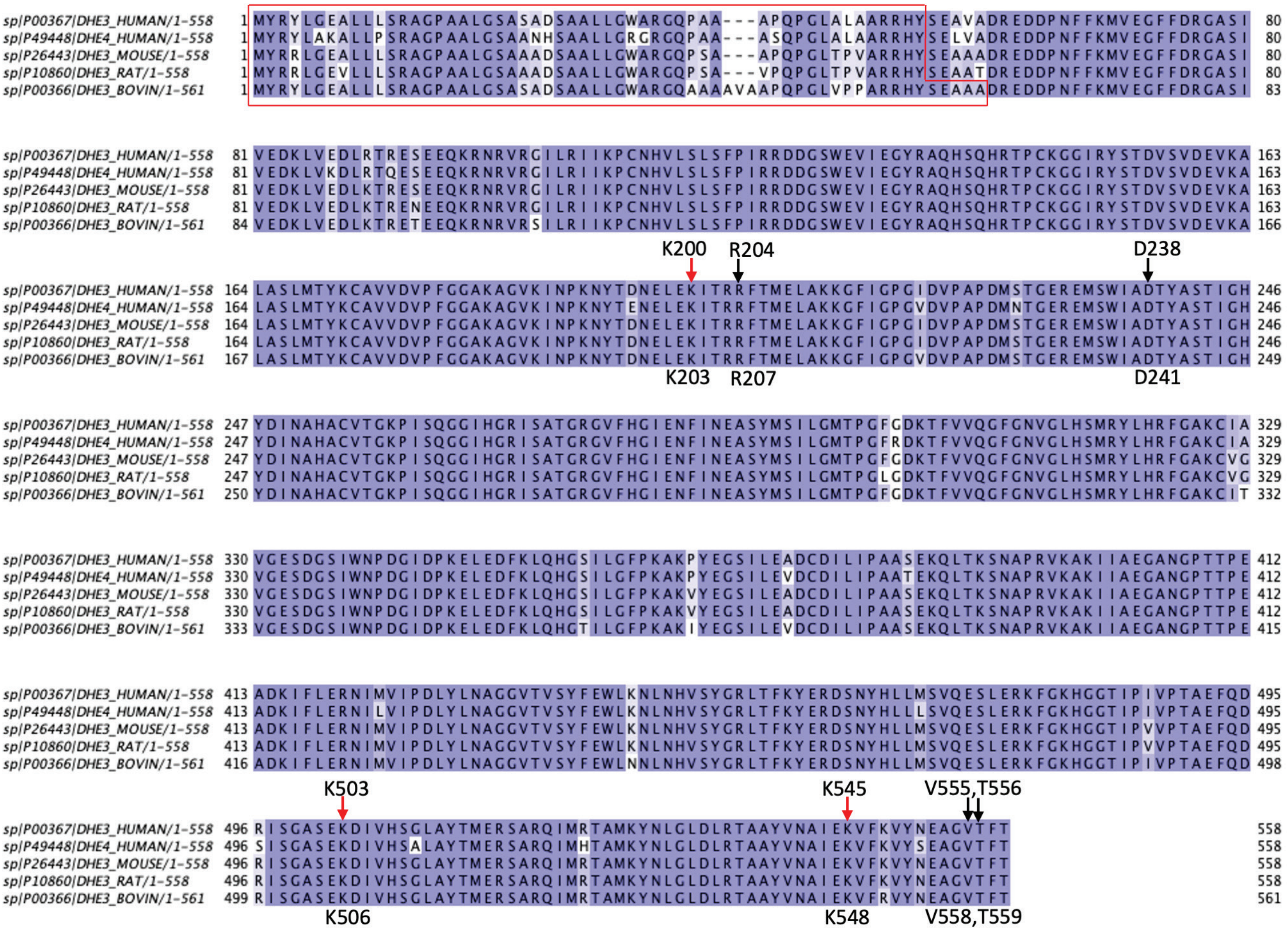

Supplementary Figure S5. Multiple alignment of the five mentioned GDH sequences: human GDH1 (DHE3_HUMAN) and GDH2 (DHE4_HUMAN), and GDH from mouse, rat and cattle, respectively. Uniprot accession numbers are provided within the sequence identifiers on the left. The N-terminal transit peptide sequences are contoured in red (top), while the residues involved in leucine binding are indicated by the black arrows. The residues discussed as susceptible to acetylation are highlighted by the red arrows. Differences in the sequences of transit peptides result in different numeration, which is shown above (as in human GDH) or below (as in bovine GDH) the indicated residues.
